# Benign posterior mediastinal schwannoma—Multiple diagnostic imaging modalities

**DOI:** 10.1002/ccr3.2274

**Published:** 2019-10-31

**Authors:** Yeo‐Jeong Song, Sang‐Hoon Seol, Seunghwan Kim, Dong‐Kie Kim, Ki‐Hun Kim, Doo‐Il Kim, Do‐Kyun Kang, Ji Yeon Kim

**Affiliations:** ^1^ Department of Internal Medicine Inje University College of Medicine, Haeundae Paik Hospital Busan Korea; ^2^ Division of Thoracic Surgery Inje University College of Medicine, Haeundae Paik Hospital Busan Korea; ^3^ Department of Pathology Inje University College of Medicine, Haeundae Paik Hospital Busan Korea

**Keywords:** diagnostic modalities, mediastinal schwannoma

## Abstract

Schwannoma is usually benign, encapsulated spindle cell tumor which arises from schwann cells of nerve sheath, and is the most common of the neurogenic mediastinal tumors. Various imaging modalities can be applied to assess posterior mediastinal mass which is often found incidentally without symptom and frequently misdiagnosed for other benign conditions both clinically and radiologically in which clinicians should be aware of.

## Graphical abstract

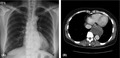

## 1 INTRODUCTION

Neural originate tumors are the most common primary mediastinal neoplasms. A large proportion of them are benign. Schwannomas are the most common type of neurogenic tumor observed in thorax which originated from the peripheral nerve sheath of Schwann cells and often located in the posterior mediastinum.^1^ It is frequently found incidentally as an asymptomatic mass by various imaging modalities.^2^ We report a case of incidental schwannoma in middle‐aged woman who was treated successfully by complete surgical resection of the mass.

## 2 CASE HISTORY

**Figure 1:**
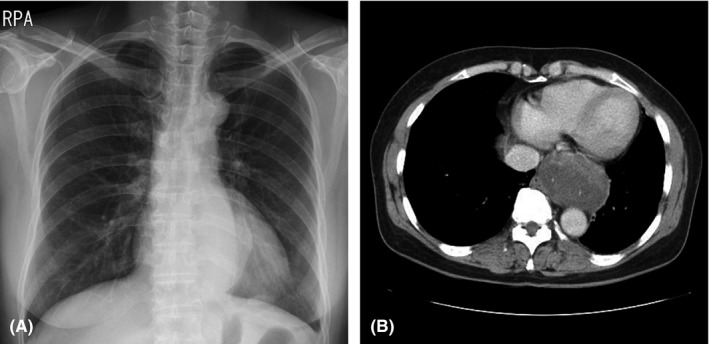
Chest X‐ray showed mediastinal mass in posterior cardia area (A). The chest computed tomography showed an oval shaped mass of 7.4 × 4.5 cm size in the posterior mediastinum. The mass compressed the left atrium, esophagus, and no definite invasion to lung (B)

**Figure 2:**
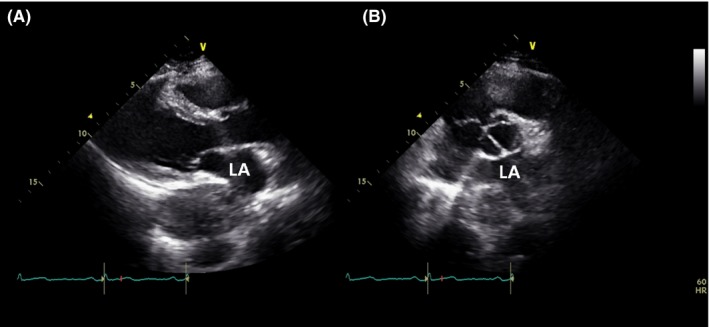
Transthoracic echocardiography revealed well‐defined heterogeneous oval mass compressing left atrium in the parasternal long (A) and short axis view (B). LA, left atrium

**Figure 3:**
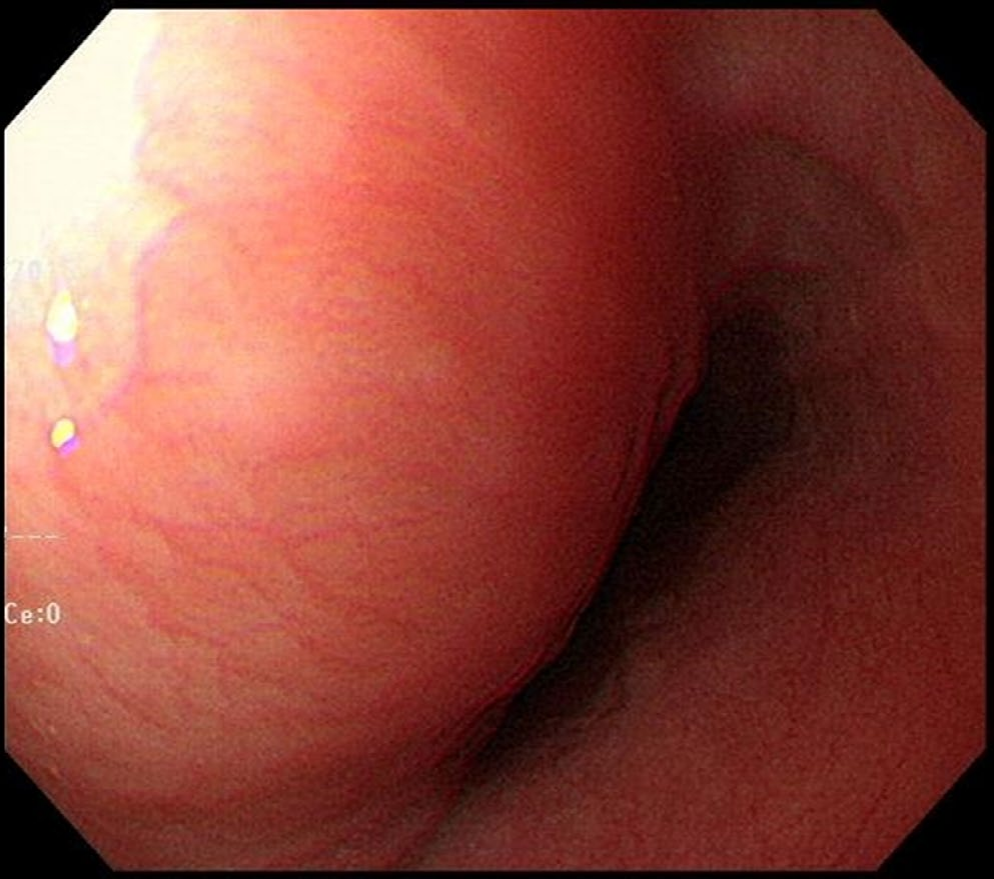
Gastrofibroscopy demonstrated a huge bulging mass at the distal esophagus

**Figure 4:**
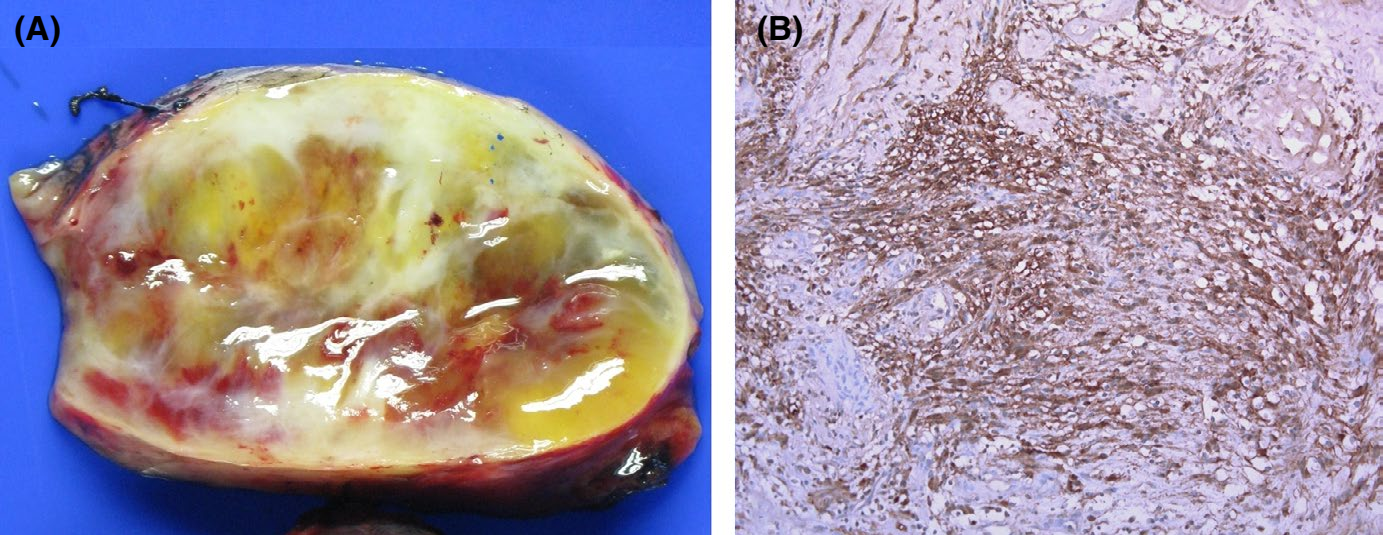
The pathology demonstrated that microscopic finding revealed white/yellow, soft mass with extensive myxoid degeneration and focal hemorrhage (A). Immunohistochemical staining showed that tumor cells were strongly positive staining for spindle cells (S100, ×200) (B)

A 60‐year‐old woman with cough was referred to our hospital for general check‐up. The patient had no pathologic symptom and underlying disease. Chest X‐ray showed an incidental mediastinal mass in posterior cardia area (Figure 1A). Transthoracic echocardiography showed well‐defined heterogeneous oval mass compressing left atrium (Figure 2). Gastroendoscopy revealed a huge bulging subepithelial mass at the distal esophagus, which was extended to mid esophagus (Figure 3). Chest computed tomography (CT) demonstrated a hypodense oval mass of 7.4 × 4.5 cm size in the posterior mediastinum. The mass compressed the left atrium, esophagus without definite invasion to lung (Figure 1B). After verifying the mass localization, the patient underwent surgical excision. The gross finding of the tumor showed well localized white and yellowish soft mass with extensive myxoid degeneration and multiple focal hemorrhages (Figure 4A). Immunohistochemical staining demonstrated the groups of spindle cells (S100, ×200) with waxy nuclei which strongly suggested a histopathologic finding of the benign schwannoma (Figure 4B). No marginal invasion was observed. The patient was eventually discharged from the hospital without any complication.

## 3 DISCUSSION

Schwannoma is the most common benign neurogenic tumor that originated from the peripheral nerve sheath of Schwann cells. Intrathoracic schwannoma is most frequently located in the posterior mediastinum.^1^ It is usually slow growing mass with low potency of malignancy. In terms of rare cases of malignant deformation, broad fusiform typed pleomorphic spindle cells may exist with necrotic and hemorrhagic changes.^3^ Alike above case, it is incidentally found as an asymptomatic mass on the imaging studies such as echocardiography, gastroendoscopy, and chest CT.^2^ Clinical symptoms may not appear until surrounding organs have been invaded or compressed by the tumor. CT may be helpful in confirming the extension and respectability of the tumor.^4^ Complete surgical excision is the treatment of choice for mediastinal schwannoma because of virtual pulmonary complications intruding intercartilaginous membrane of trachea causing hemoptysis, dyspnea, or chest pain.^2^ The other complication may involve gastrointestinal bleeding, cardiac tamponade, and dysphonia in severe cases. Approximately 10% of the cases may invade intervertebral foramen which results in the compression of vertebral canal.^5^ Fortunately, the successful complete surgical resection was done in above patient without any need of adjuvant chemotherapy. Various imaging modalities can be applied to assess posterior mediastinal mass which is often found incidentally without symptom and frequently misdiagnosed for other benign conditions in which clinicians should take heed of.

## CONFLICT OF INTEREST

None declared.

## AUTHOR CONTRIBUTIONS

YJS: involved in literature search and the writing of the manuscript. SHS: investigated and supervised the overall work. SK, DKK, KHK, and DIK: discussed the results and advised about the case. Do‐Kyun K: performed the operation and provided surgical information. JYK: provided pathological information and images.
